# Gene mutation associated with *esl* mediates shifts on fungal community composition in rhizosphere soil of rice at grain-filling stage

**DOI:** 10.1038/s41598-018-35578-y

**Published:** 2018-11-30

**Authors:** Puleng Letuma, Yasir Arafat, Muhammad Waqas, Feifan Lin, Weiwei Lin, Yiyang Zhang, Mamello Masita, Kai Fan, Zhaowei Li, Wenxiong Lin

**Affiliations:** 1 College of Crop Science, Fujian Agriculture and Forestry University, Fuzhou, 350002 Fujian, China; 2 Key Laboratory for Genetics, Breeding and Multiple Utilization of Crops, Ministry of Education, Fuzhou, China; 3 Fujian Provincial Key Laboratory of Agroecological Processing and Safety Monitoring, Fujian Agriculture and Forestry University, Fuzhou, China; 4 College of Life Sciences, Fujian Agriculture and Forestry University, Fuzhou, China; 5 Crop Science Department, Faculty of Agriculture, National University of Lesotho, Roma, 180 Lesotho

## Abstract

Generally, plant roots shape the rhizosphere fungal community but how individual plant genes involved in senescence affect this shaping is less studied. We used an early senescence leaf (*esl*) mutant rice and compared it with its isogenic wild type variety to evaluate the effect of the vacuolar H^+^-ATPase (VHA-A1) gene mutation on the rhizosphere fungal community structure and composition using a metagenomic pyrosequencing approach. The most predominate fungal phyla identified for both isogenic lines belonged to *Ascomycota*, *Basidiomycota* and *Glomeromycota*, where *Ascomycota* were more prevalent in the *esl* mutant than the wild type variety. Real-time quantitative PCR analysis confirmed a significant rise in the richness of *Cladosporium cladosporioides* in *esl* mutant rice than the wild type variety. Correlation analysis revealed four most abundant genera identified for the *esl* mutant and their close association with yield and biomass decline, lipid peroxidation, lower root vitality, chlorophyll degradation and limited VHA activity. Higher K^+^ efflux, H^+^ and a lower Ca^2+^ influx was also observed in the *esl* mutant which could be the reason for abnormal functioning of mutant plants. These results illustrate that besides the well-known effect of senescence on plant physiology and yield decline, it can further shape the rhizosphere fungal community.

## Introduction

Rice (*Oryza sativa* L.) is the main staple food consumed by more than half of the world population, with more than 480 million metric tons produced annually^1^. Hybrid rice varieties have been developed to overcome biotic and abiotic stresses and increase the yield. However, a major constraint in hybrid rice production that can result in yield reduction thereby threatening food security is the timing of leaf senescence during the grain filling stage^2^. There are three possible reasons for the substantial decline in yield and grain quality owing to the timing of senescence: (i) reduction in biomass accumulation via photosynthetic activity termination, (ii) nutrient remobilization from senescing tissues to actively growing parts furthermore, (iii) senescence associated with environmental stress that causes yield reduction as well as lower nutrition status^3,4^.

In cereals, leaf senescence is characterized by nutrient remobilization, chlorophyll degradation, and sometimes death of tissues^3^. Chlorophyll degradation, which is a familiar phenomenon in the leaf senescence program, is accompanied by a decrease in photosynthetic activity, chloroplast structural loss, protein degradation, lipid membrane peroxidation, and malondialdehyde (MDA) buildup^5,6^. This physiological set of events linked with senescence is prompted by the reprograming of many genes, down- or up-regulated, in response to definite senescence-promoting elements. Previous studies have reported the ability of biotic and abiotic stress that speed up leaf senescence^7^.

Several studies indicate how abiotic stresses trigger leaf senescence by reprogramming certain subsets of senescence-associated genes (SAGs) that are differentially expressed in distinct tissues^3^. However, information about the relationship between leaf senescence and biotic factors is limited, especially with respect to leaf senescence related to rhizosphere microbes. Previous research has shown that plant roots are in complex interaction with soil microbial communities in which both biotic and abiotic factors play major roles in shaping the microbial communities in soil^8–11^. Moreover, it has been indicated that the rhizosphere where plant roots interact is a plant nutrient enhanced niche is extremely competitive environment that also influences plant root health^12,13^.

Recently, attention has been given to the soil biodiversity, particularly plant-microbe communications^14,15^. Evidence demonstrates that the key plant and microbial traits can disturb plant nutrition, health, and yield^16,17^, for instance, fungi are abundant micro-organisms, which perform vital role in the soil ecology as main decomposers of organic matter and release nutrients through nutrient cycling thus promote plant development in soils^15,18^. Therefore our study seems to be relevant as some fungi can destroy the whole agricultural field by causing plant diseases^19^, while others have antagonistic capabilities towards plant pathogens^15,20^.

Recent investigations have indicated that plant genotype also has a great influence on the structure of root microbiomes, implying the role of the host in the construction of microbial communities^21–25^. On the other hand, previous researches using high-throughput investigation revealed that the host genotype had small but important effect on the microbial composition in *Arabidopsis thaliana* plant roots^26,27^. However, other studies reported that plant developmental stages also alter the rhizosphere microbial population^28,29^ more than the host genotype itself.

It is well documented that transport through the tonoplast (across membranes) is energized by two proton pumps, the vacuolar H^+^-ATPase (V-ATPase) and the vacuolar H^+^-pyrophosphatase (V-PPase)^30^. The collective activity of the two pumps generates the proton gradient and the membrane potential for transport by hydrogen ion gradient driven symport of compounds against their concentration gradients^31^. Additionally, a physiological role of V-ATPase has been noted in seed germination^32^, environmental stress tolerance^33^ and in plant growth control^34^. Because of its important role in plant physiological processes, substantial evidence indicate that a deletion of V-ATPase induced seed dormancy and early senescence in rice plants^35^.

Despite several studies examining premature senescence in leaves, the fundamental mechanism remains mostly unknown due to its complexity. A deeper understanding of soil microbial community composition and diversity coupled with the use of senescence-associated mutants can help improve our understanding of senescence mechanism at a late developmental phase in economically important crops. However, to our knowledge there is no information presented on how premature senescence influences the rhizosphere microbial communities at grain-filling stage. We hypothesized that plant modifications influencing the quality and quantity of resources available for microorganisms could reveal some of the observed variations in rhizospheric fungal communities. Hence the objective of this study was to examine the effects of a vacuolar H^+^-ATPase (VHA-A1) gene mutation in rice expressing early senescence leaf (*esl*) on the rhizosphere fungal community composition and diversity. Our study was aimed to clarify the underlying mechanism of early senescence that occurs in *esl* mutant rice using pyrosequencing approach. Moreover, we performed correlation analysis to determine how the identified fungal communities and physiological traits related to early senescence.

## Results

### Soil chemical properties

The soil chemical properties were detected at grain-filling stage to determine the principal causes of senescence in rice isogenic lines in the field (Supplementary Table 1). All the soil nutrients (NPK) did not show a significant difference between the *esl* mutant rice and its isogenic variety. The total nitrogen of 1.06 and 1.05 g kg^−1^; available nitrogen of 14.65 and 14.68 mg kg ^−1^; total phosphorus of 26.72 and 26.65 g kg^−1^; available phosphorus of 101.67 and 95.00 mg kg^−1^; total potassium of 11.31 and 11.33 g kg^−1^; and available potassium of 131.65 and 134.34 mg kg^−1^ were recorded for wild type and *esl* mutant, respectively. Moreover, soil pH was not significantly different for wild type (5.4467) and *esl* mutant (4.89) soil samples.

### The biomass, yield and physiological traits of *esl* mutant rice and its isogenic wild type variety

Root to shoot ratio was determined from dry shoot and root biomass (Supplementary Fig. 1), a higher biomass, panicle number per plant, filled grain and percentage filled grain were recorded 150 days after transplanting for a wild type variety than a mutant (Table 1). A notable yield reduction in *esl* mutant (16.10 g) than the wild type variety (26.587 g) was observed probably due to early senescence. A similar pattern occurred in chlorophyll content and root oxidizability (% R.O.) estimated at 30 days after flowering (DAF). However, MDA and electrolyte leakage (E.L. %) were significantly higher than those observed for a mutant than the wild type variety, with the effect more pronounced in roots than the leaves (Table 1). Our results also revealed a higher vacuolar H^+^-ATPase (VHA) activity in both leaves and roots for a wild type variety than the *esl* mutant.

**Table 1:** Effect of senescence on rice biomass, yield and physiological parameters of *esl* mutant rice (Mut) and its isogenic wild type (WT) variety.

Parameter	WT	Mut
Root to shoot ratio	0.2519a	0.1937b
Panicle number/plant	14.667a	6.33b
1,000-seed weight (g)	26.587a	16.10b
Filled grain (g)	2679.3a	364.7b
% filled grain	88.133a	73.667b
Chlorophyll content	43.883a	30.933b
Electrolyte leakage % (r)	44.93b	67.10a
Electrolyte leakage % (l)	20.37b	30.93a
MDA(r) (nmol g^−1^ FW)	0.1364b	0.2284a
MDA(l) (nmol g^−1^ FW)	0.1713b	0.2392a
% Root oxidizability	38.54a	26.65b
VHA activity (r) (µmol pi mg^−1^ protein h^−1^)	0.3845a	0.2974b
VHA activity (l) (µmol pi mg ^−1^ protein h^−1^)	0.4152a	0.293b

Means followed by different letters in a row are significantly different (P ≤ 0.05, n = 4). *Significant at 1% level of probability. *Significant at 5% level of probability; Tchl, Total chlorophyll; MDA(r), (l), Malondialdehyde in roots and leaves, respectively; E.L. % (r), (l), electrolyte leakage percentage in roots and leaves respectively; % R.O., percentage Root oxidizability; VHA (r), (l), Vacuolar H^+^-ATPase activity in roots and leaves, respectively.

### The 18S rRNA amplicon metagenomics analysis of soil fungus

**Figure 1:**
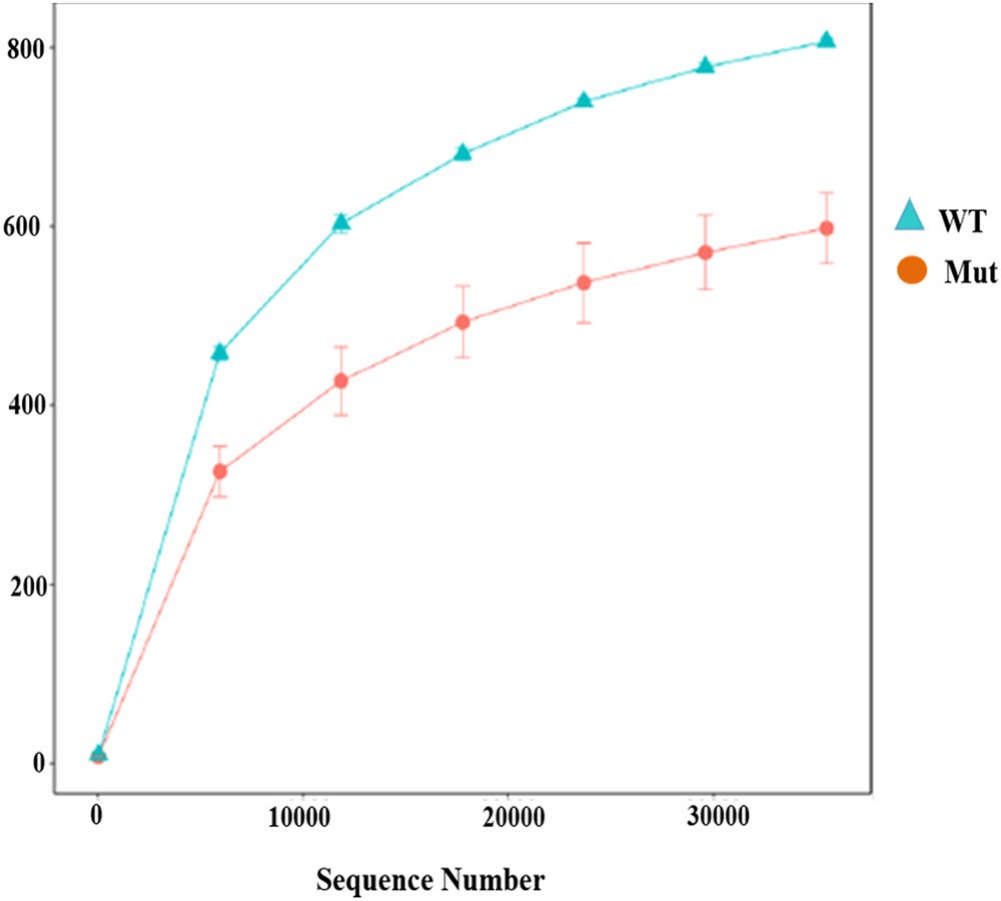
Rarefaction curves of fungal communities based on observed OTUs at 3% dissimilarity for soil samples.

We analyzed the rhizosphere community by 454 pyrosequencing and generated 311,764 high quality 18S rRNA sequences. After quality filtering, a total of 5,301 paired-end reads were obtained from six rhizosphere samples. The sequencing coverage reached 98%, this suggested that the sequence depth was sufficient to capture the relative diversity and richness of these samples accurately. On the other hand, the average length of these reads was 324 bp. All reads were clustered into 4,563 operational taxonomic units (OTUs) having 1,637 unique tags at 3% distance dissimilarity (Supplementary Tables 2 and 3). To evaluate the diversity and richness of the six samples we used rarefaction curves. The analysis displayed that the quantity of species detected tended to plateau at 30,000 sequences, with considerable higher richness in wild type than in mutant samples (Fig. 1).

### Fungal alpha-diversity

**Figure 2:**
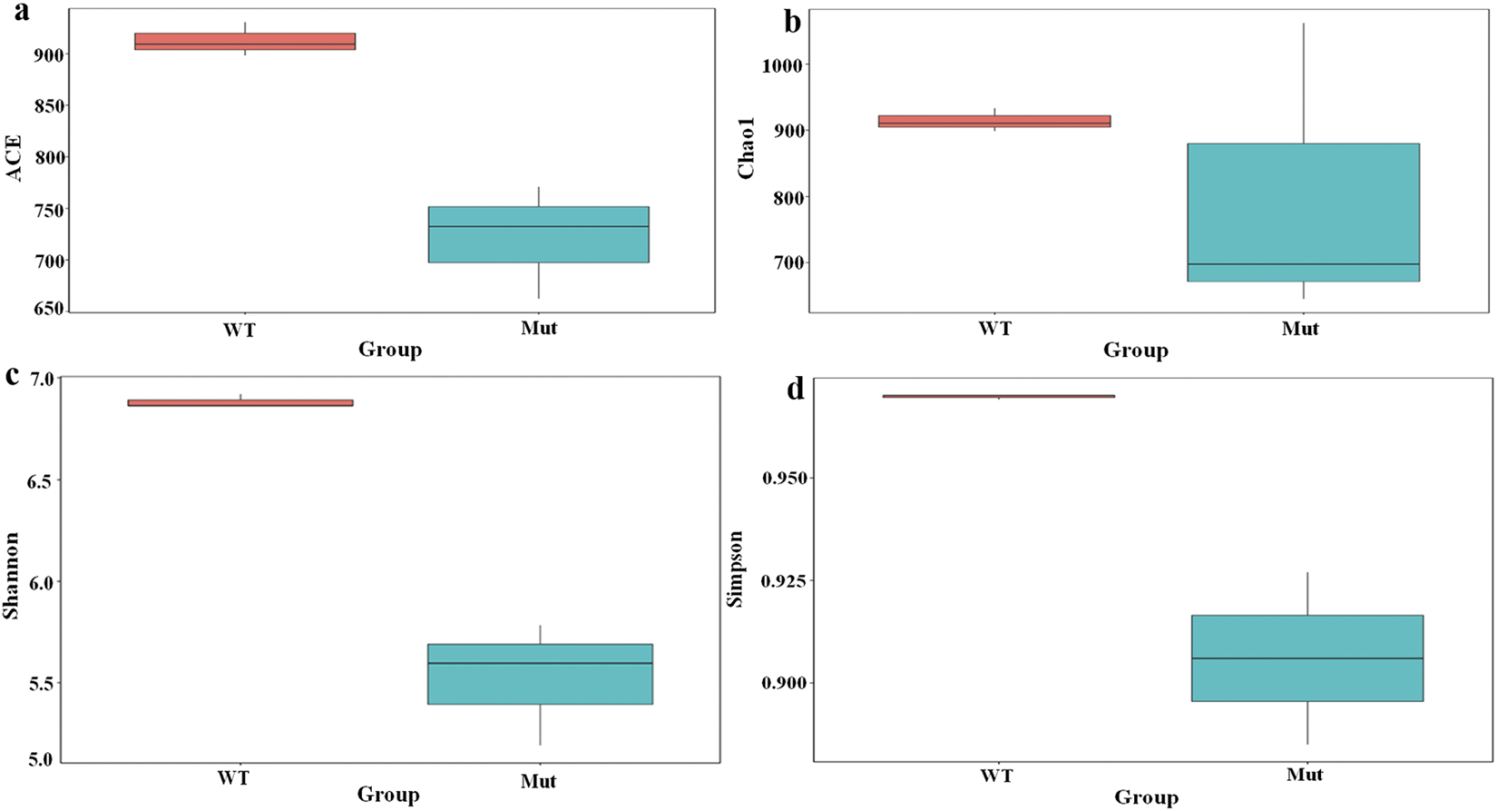
Estimates of α-diversity indices including (**a**) abundance-based coverage estimators (ACE), (**b**) Chao1, (**c**) Shannon, and (**d**) Simpson in wild type (WT) and *esl* mutant (Mut) soil samples.

The richness and diversity of the sequenced data for wild type and mutant soil samples is illustrated in Fig. 2. Observed species, Shannon, Simpson and Chao1 estimator were higher in wild type variety than the mutant. The level of coverage presented here demonstrates that the 18S rRNA sequences identified in these samples represent most fungal sequences. Moreover, the number of OTUs identified was close to the total number of OTUs assessed by Chao1 and ACE diversity indices examined at 3% dissimilarity in both samples, thus suggesting that the fungal communities were well covered (Supplementary Table 2).

### Variation in fungal community structure

**Figure 3:**
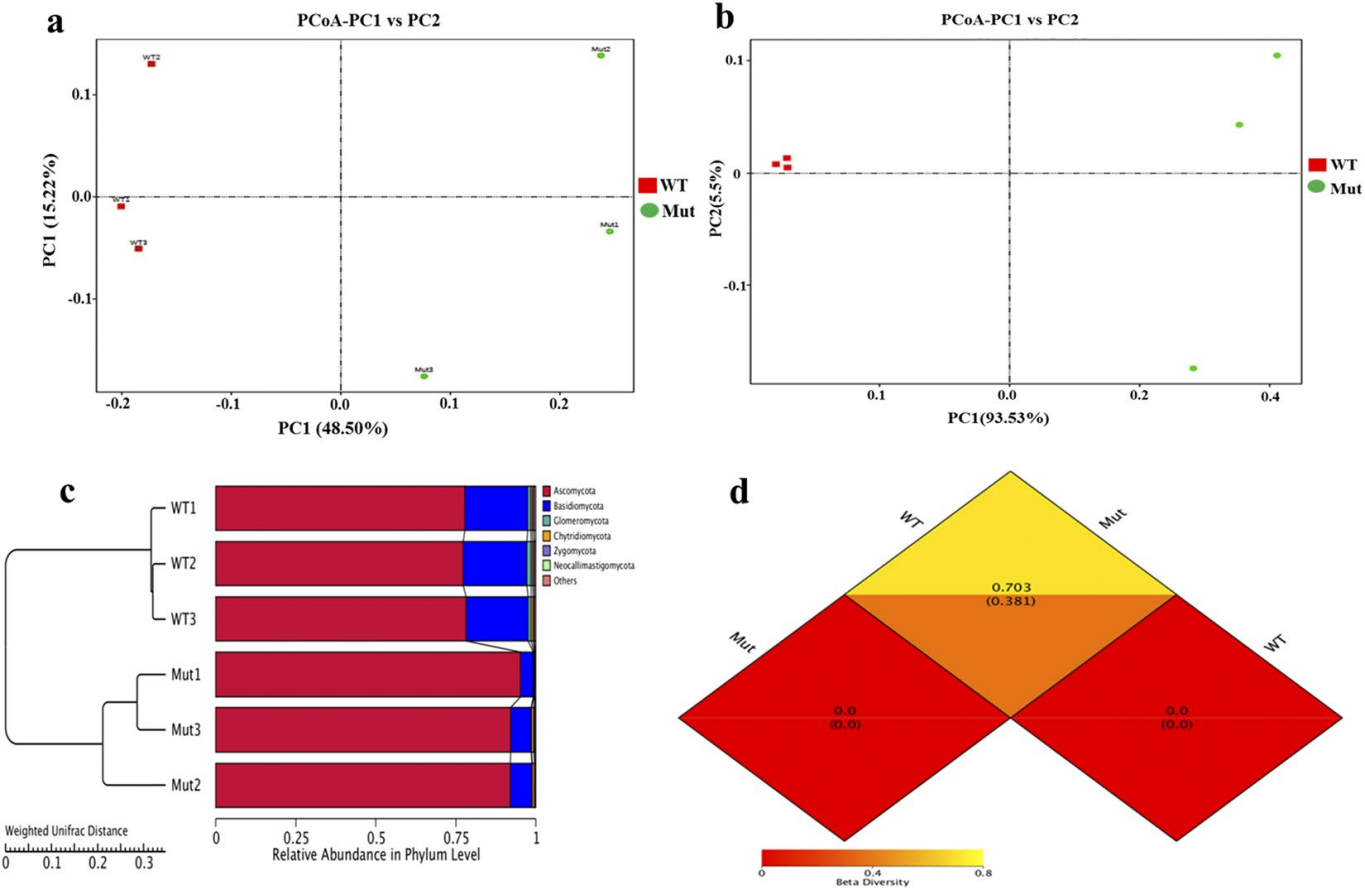
Biplot ordination of (**a**) UniFrac-weighted principal coordinate analysis (PCoA) and (**b**) Unweighted UniFrac (UUF) PCoA for the tested soil samples, WT represents an isogenic wild type variety, while Mut represents *esl* mutant. (**c**) UPGMA/hierarchical clustering analysis based on weighted UniFrac distances displaying the relative abundance of the most abundant fungal phylum of WT and Mut soil samples. (**d**) β-diversity heat map based on weighted (WUF) and unweighted (UUF) UniFrac distances. Values in the upper and lower corners represented the WUF and UUF distances.

**Figure 4:**
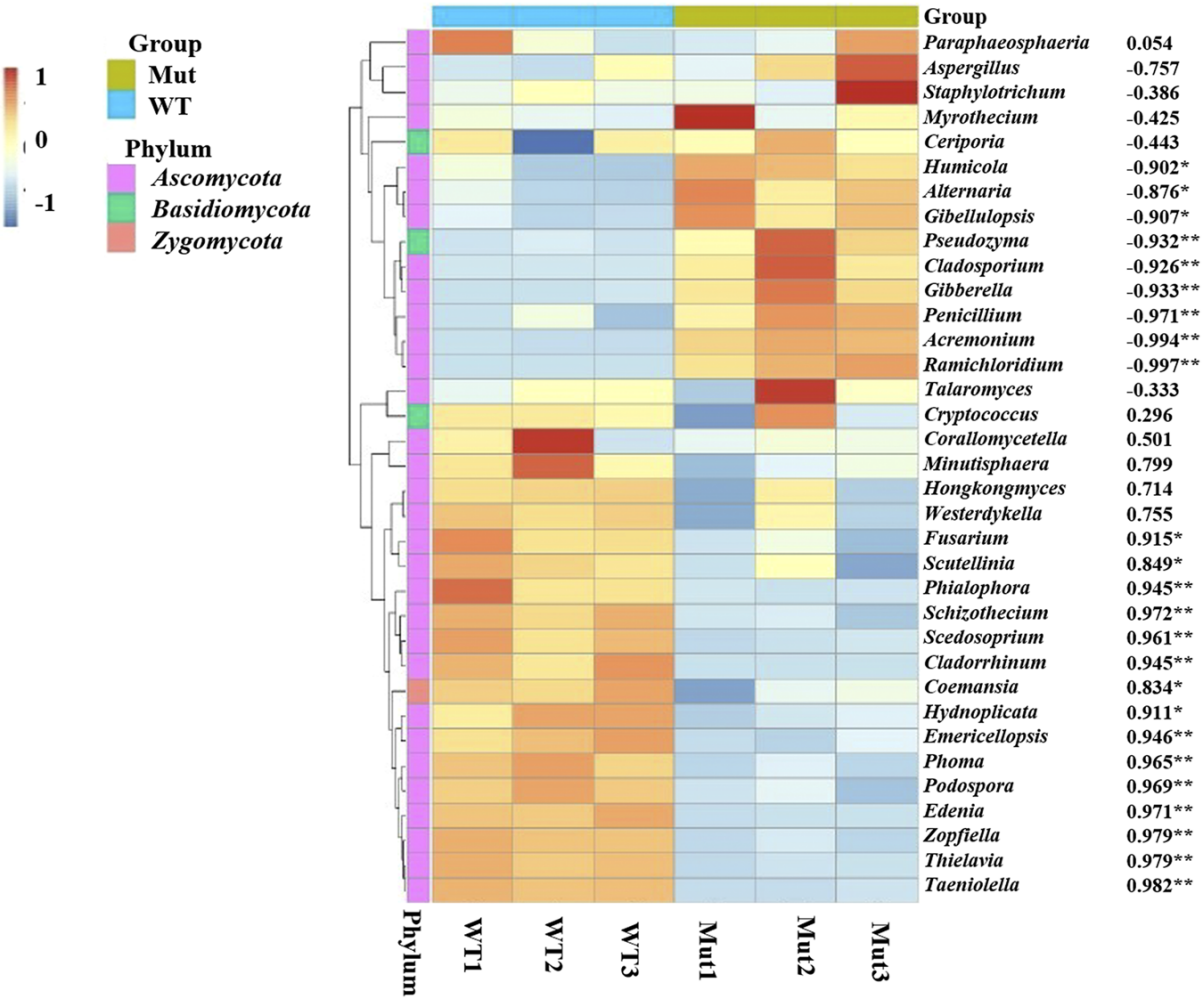
Heat map displaying the distribution of the 35 most abundant fungal genera and their Pearson’s correlation coefficients with yield (1,000-seed weight). Data are mean values of n = 3; significant correlation coefficients are noted in asterisks where P ≤ 0.05.

We used weighted Unifrac distance for principal coordinate analysis (PCoA) to determine how dissimilar the soil fungal communities were between the wild type variety and an *esl* rice mutant. The rhizospheric fungal community of wild type variety was distinctly different from the one of an *esl* mutant (Fig. 3a). The variation was observed through analysis of scatter plots, where the scatter distributions of the wild type were similar and those of a mutant revealed a significant difference. The first and second principal components accounted for 93.53% and 5.51%, respectively of the total variation in fungal communities among the individual samples. This data clearly indicates that microbial communities found in the wild type variety is significantly different from those in the mutant samples. In addition, the hierarchical cluster analysis indicated distinct differences in fungal community structure among the two treatments and similar arrangements for the same samples when evaluated in three replications (Fig. 3c). PCoA results alluded with those of heat map study, which revealed that all the samples presented a variation in fungal communities (Figs 3a and 4). Further, unweighted UniFrac PCoA examination indicated that the fungal communities in the *esl* mutant rhizospheric soil were distinctly dissimilar from those of the wild type variety. Both mutant and wild type samples were well assembled along the principle coordinate axis-1 (93.54% of variation) while mutant samples were spread out along axis-2 (5.5% variation) (Fig. 3b). The weighted pair group method with arithmetic mean clustering analysis further revealed the variability between the samples analyzed in triplicate (Fig. 3c). We further used the weighted UniFrac and unweighted Unifrac distance matrices between the samples and we observed a wider distance between the samples with 0.703 and 0.381, respectively for wild type variety and *esl* mutant (Fig. 3d).

### Fungal abundance analysis at phylum and genus level

The OTUs were aligned with the SILVA database and then grouped into phylotypes comprising of six phyla (Supplementary Fig. 2). It is noteworthy that the relative abundance of *Ascomycota* was significantly higher for mutant than the wild type, accounting for 93.13% and 77.78%, respectively (Supplementary Table 4). On the other hand, the abundance of *Basidiomycota* was highest for wild type variety (19.6%) than mutant (5.67%). Similarly, we detected an upturn in the abundance of *Glomeromycota* (1.1%) in the wild type compared to 0.18% in the mutant.

To further verify that microbial communities were different between the samples we used a hierarchical clustering heat map analysis at the genus level constructed from the highest 35 most abundant fungal communities across all six samples (Fig. 4). In the current study, it was noted that the genus *Cladosporium* clearly dominated in mutant than in wild type soil samples and was negatively correlated to yield (R^2^ = −0.926, *P* ≤ 0.01), surprisingly, *Fusarium* was positively associated with 1,000-seed weight (R^2^ = 0.915, *P* ≤ 0.05). It was further observed that the relative abundance of *Cladosporium* and *Thielavia* sequences was higher, accounting for 4.9% and 4.3% contribution to the dissimilarity, respectively (Table 2).

**Table 2:** Top genera with 50% cumulative contribution to the dissimilarity amongst the samples.

Genus	Class	Phylum	% Contribution	% Relative Abundance	
				WT	Mut
Cladosporium	Dothidiomycetes	Ascomycota	4.9	0.3000b	9.5667a
Thielavia	Sordoriomycetes	Ascomycota	4.3	6.300a	2.300b
Zopfiella	Sordoriomycetes	Ascomycota	3.8	5.900a	1.8b
Giberella	Sordoriomycetes	Ascomycota	1.9	0.3333b	3.4667a
Fusarium	Sordoriomycetes	Ascomycota	1.3	1.6333a	0.9333b
Westerdykella	Dothidiomycetes	Ascomycota	1.3	1.7000a	1.000b
Hongkongmyces	Dothidiomycetes	Ascomycota	1.3	1.600a	1a
Taeniolla	Dothidiomycetes	Ascomycota	1.1	2.033a	0.2333b
Humicola	Sordoriomycetes	Ascomycota	1.0	0.8667b	1.2333a

Means followed by different letters in a row are significantly different (P ≤ 0.05, n = 3).

### Shared fungal communities by different sample groups

**Figure 5:**
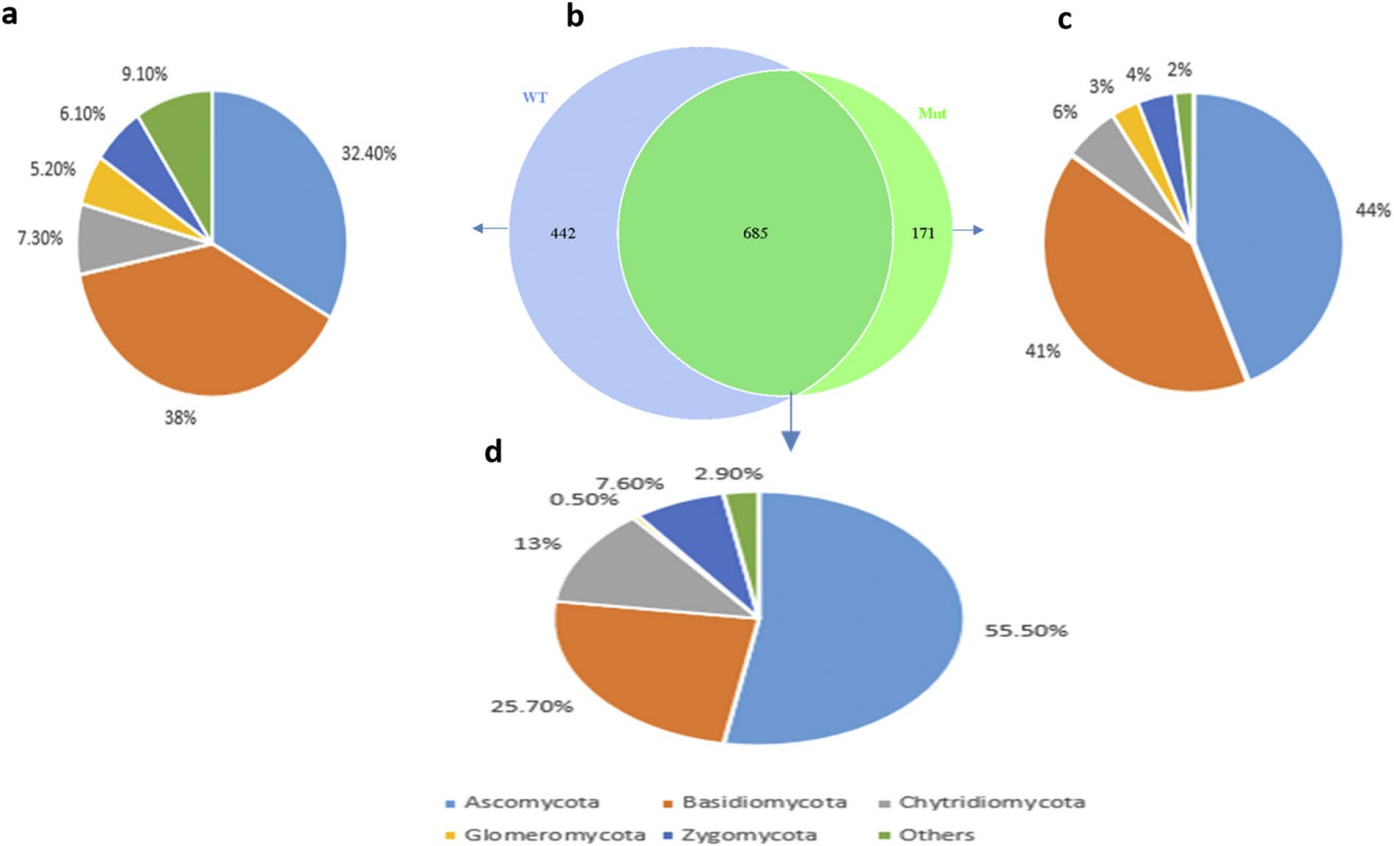
Venn diagram showing the number of shared and unique OTUs in different sample groups. (**a**) Unique OTUs (442) in the samples of wild type rhizhosphere soil (**b**) Venn diagram figure for fungal OTUs in the two rhizospheric soil samples (**c**) Unique OTUs (171) in the samples of *esl* mutant rice (Mut) rhizosphere soil. (**d**) OTUs (685) shared by mutant and wild type variety at grain-filling stage.

To understand the distribution of OTUs among the two samples the sequences of all samples were grouped together. The generated Venn diagrams indicated special and common OTUs among the samples, with most of the species belonging to the phylum *Ascomycota* (Fig. 5). The results displayed that 685 OTUs were common in both samples (Fig. 5b,d), this indicates a similar community structure between the wild type variety and the mutant. Conversely, there were a few OTUs (442) that were only present in wild type samples only (Fig. 5a) and mutant samples had fewer distinctive OTUs (171) compared to its isogenic wild type variety (Fig. 5c). *Ascomycota* was the most dominant taxa with 55% and 53% for shared OTUs and mutant (Mut) samples, respectively. Nevertheless, the abundant taxa for wild type (WT) samples were *Basidiomycota* with 38% followed by *Ascomycota* with 32.4%.

### Senescence modulated K^+^/H^+^ by increasing K^+^ and H^+^ loss and decreasing the Ca^2+^

**Figure 6:**
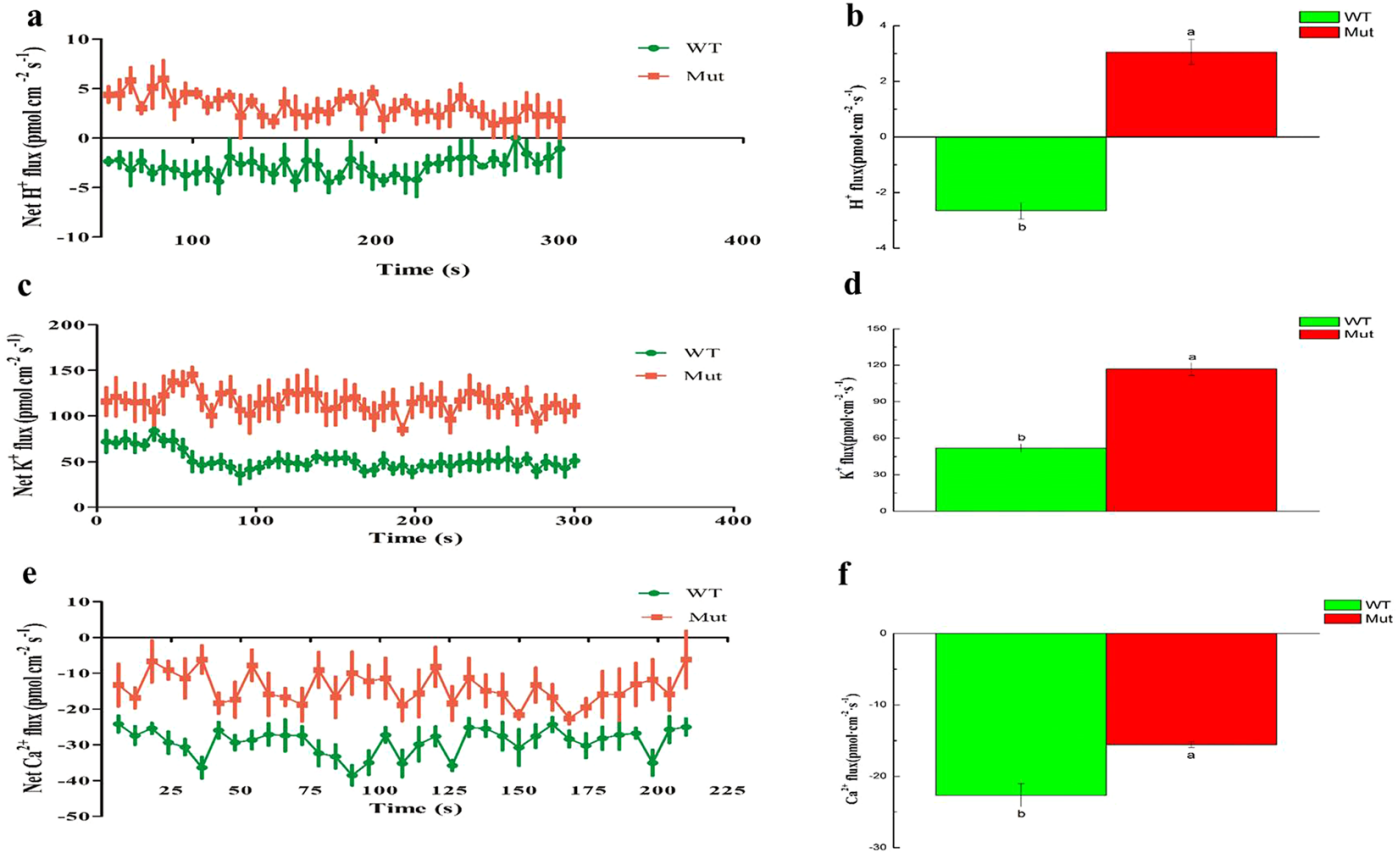
The net H^+^, Ca^2+^ and K^+^ flux in rice roots from *esl* mutant and the wild type variety at grain-filling stage. The positive values in the figures represent the net efflux and negative value represents the net influx. Each point represents the mean value of four individual roots and bars represent the SE of the mean. The mean fluxes of H^+^, K^+^ and Ca^2+^ within the measuring periods are shown alongside and different letters indicates a significant difference at P ≤ 0.05.

To clarify the role of VHA and intracellular ionic balance, we measured the activity of VHA and the net Ca^2+^, H^+^, and K^+^ fluxes for the two genotypes at grain-filling stage. The activity of VHA in rice roots and leaves were determined for *esl* mutant rice and its isogenic wild type variety. The *esl* mutant showed significantly lower activities (roots and leaves) than the wild type variety (Table 1). Non-invasive Micro-Test Technology (NMT) results showed a two-fold H^+^ efflux (Fig. 6a,b) and a higher K^+^ efflux in *esl* mutant than the wild type roots (Fig. 6c,d), implying that mutant plants had higher H^+^ and K^+^ ion flow out of the root than the wild type plants. However, the opposite trend was recorded for Ca^2+^ which was negative but significantly higher for the wild type variety (−22.308, P ≤ 0.05) than the mutant (−16.552, P ≤ 0.05) (Fig. 6e,f).

### Correlation between fungal communities, plant biomass, yield and physiological traits

We used Pearson’s correlation to evaluate relationships between yield related traits and abundant genera (Supplementary Table 5). Interestingly, all the abundant genera in an *esl* mutant rice were significantly and negatively correlated to plant biomass and yield related traits. In general, fungal communities identified in the wild type variety were positively correlated to plant biomass and yield related traits, for instance *Thielavia* and 1,000-seed weight (R^2^ = 0.971, P ≤ 0.01).

To determine what fungal taxa were associated with physiological parameters between the isogenic lines, Pearson’s correlation was performed. The analysis revealed that relative abundance of *Cladosporium, Gibberella, Humicola* and *Penicillium* were negatively correlated to total chlorophyll, % R.O. and VHA activity; on the other hand they were positively correlated to MDA and E.L. % in both roots and leaves (Supplementary Table 6). On contrary, abundant fungal communities identified associating with the wild type variety were positively correlated to all the parameters mentioned above and negatively correlated to MDA and E.L. levels. Likewise, Pearson’s correlation was used to evaluate relationships between biomass, yield and physiological traits (Supplementary Table 7). The results indicated that plant biomass and yield were positively correlated to chlorophyll content, % R.O. and the activity of VHA. Interestingly, the stress indicators MDA and electrolyte leakage on both roots and leaves were strongly but negatively associated with biomass and yield (Supplementary Table 7).

### Abundance of *Cladosporium cladosporioides* by RT-PCR

Firstly, we developed a standard curve of y = 0.2741x + 9.8701 (R^2^ = 0.999) for *C. cladosporioides* by real-time PCR (RT-PCR) analyses. The abundance of *C. cladosporioides* was significantly (*P* ≤ 0.05) higher in *esl* mutant rice soil samples (Mut) than in wild type variety (WT). The RT-PCR results were consistent with the pyrosequencing results for this specie in *esl* mutant rice than the wild type variety (Supplementary Fig. 3).

## Discussion

Leaf senescence impacts crop economic yield and grain quality important for food and feed industry. In the present study, we shed light on the effect of the vacuolar H^+^-ATPase (VHA-A1) gene mutation on the rhizosphere fungal community structure and composition using a metagenomic pyrosequencing method, using two rice isogenic lines, one with early senescence leaf mutant (*esl*) phenotype and its wild type variety. Schappe *et al*.^36^ reported that soil chemical properties are linked to changes in rhizosphere fungal community, however in our study the soil properties between the two isogenic lines were not significantly different. This implies that the shift in rhizosphere fungal community was not related to soil chemical properties. We observed a lower root to shoot ratio for an *esl* mutant than the wild type variety, similar results were recorded in wheat lines with contrasting timing of senescence^37^. In crops, inappropriately timed senescence can lead to yield decline, particularly if it occurs at grain-filling stage^2^. Our study revealed a yield decline in *esl* mutant rice relative to its isogenic variety (Table 1), the yield data further substantiates the fact that VHA-A1 gene mutation in the *esl* mutant is responsible for low yields observed in the mutant than in the wild type variety.

In the present study, chlorophyll content, MDA concentration, % R.O. and E.L. % were regarded as the cellular indicators of leaf senescence. Our study revealed lower chlorophyll content and a higher MDA and E.L. in *esl* mutant, suggesting a more senescent type damage to tissues of *esl* mutant than the wild type variety. Accordingly, Cao *et al*.^38^, Nahar *et al*.^39^ and Li *et al*.^40^ reported that MDA and E.L contents in plant tissues are indicators of lipid peroxidation and loss of cellular membranes and membrane integrity, hence why enhanced levels of MDA and E.L. were observed in *esl* mutant than its isogenic wild type variety with the effect more pronounced in roots than in leaves. Increased levels of lipid peroxidation can damage photosynthetic pigment apparatus^41^. This was presumably responsible for the lower pigment contents in the *esl* mutant than the wild type variety. Similarly, root oxidizability is regarded as the best measure of root senescence and plant vitality^42^, we also observed a lower % R.O. in the *esl* mutant than the wild type suggesting that root vitality was reduced due to root senescence. Moreover, VHA activity was lower for the mutant than the wild type variety, thus further substantiating the importance of vacuolar H^+^-ATPase in maintaining plant vitality. Our results are consistent with those previously reported by Krebs *et al*.^30^ and Tang *et al*.^43^ in which *Arabidopsis* double mutant lost 85% VHA, showing stunted growth and developed necrotic lesions. These factors are assumed to have eventually led to reduced biomass production in terms of root to shoot ratio thereby reducing the ultimate yield in the early senescence mutant than its isogenic wild type variety.

Plants can adjust their rhizosphere microbial population in a host-dependent manner where each plant species stimulates a group of selected rhizosphere microbes^13,21,44^. A study on different genotypes of maize differing in their root associated microbiome composition was partly explained by the genotype differences^22^. Contrary to this finding, Hannula *et al*.^45^ and Wang *et al*.^46^ indicated that crop cultivars had minor influence on the composition and function of fungal communities; instead the plant growth stage, year and agricultural management had a major effect on the shift in microbial composition. Our data clearly shows that fungal communities in the rhizosphere soil of wild type varies from those in *esl* samples. In the present study, we observed a significant α and β diversity between the tested genotypes, with wild type revealing the greatest microbial diversity than the *esl* mutant. Our results illustrate a clear separation between the two genotypes in terms of the abundance of their fungal communities. We therefore consider these variations were mainly due to the plant genotypes differences.

Recently, community structure and diversity of soil fungi were considered key factors of importance for soil ecosystems functions such as decomposition of organic matter, nutrient cycling, disease suppression and water dynamics^16,19,47^. From the metadata presented here, we depicted the dominance of OTUs assigned to *Ascomycota* over *Basidiomycota* and *Glomeromycota* in the mutant than wild type soil samples (Fig. 5c), proposing that senescence syndrome favors an assemblage of *Ascomycota* with the possible suppression of other groups. These findings are in accordance with those reported earlier for other agroecosystems^19,48,49^, suggesting that the composition of different fungal phyla in the rhizosphere soil samples could be similar for most cultivated plants. Moreover, a recent study on fungal communities structured by senescence revealed that *Ascomycota* were active throughout the senescence gradient, while *Basidiomycota* were present and active in senescing and dead tissues^50^.

Heat map analysis indicated that most of the core microbiome in the rhizosphere contained genera such as *Cladosporium*, *Thielavia, Zopfiella, Gibberella, Taeniolella, Fusarium, Hongkongmyces, Westerdykella, Talaromyces, Phoma, Edenia, Humicola*, and *Penicillium*, of these identified genera *Cladosporium, Gibberella, Humicola* and *Penicillium* were associated with the *esl* mutant. Our study suggests a specificity of a plant genotype for groups of fungi^48^. Recently, it has been reported that a group of microbes was stimulated and multiplied significantly, thereby creating competition with other species through exuding antimicrobial substances to inhibit the colonization of other bacterial species in cotton grown soils^51^. Similarly, the senescence responsive genera identified here could possibly benefit the plant through providing overall fitness against pathogenic attack by microbes while at the same time conferring their tolerance to senescence and other environmental stresses.

Intracellular ionic balance is closely related to the activity of VHA and hence important for cell physiological and normal functioning of the plant. Our results are in agreement with those previously reported where K^+^ influx in yeast favored survival and K^+^ efflux promoted death^52^. We observed a higher K^+^ efflux in the mutant than the wild type variety, and therefore assumed that the low K^+^ efflux, H^+^ influx and greater Ca^2+^ influx observed in the wild type variety could be the possible reasons for survival. Moreover, our study revealed a lower VHA activity in *esl* mutant than its isogenic variety suggesting that VHA-A1 deletion in *esl* mutants is the major contributing factor for low activities observed in these plants. Furthermore, earlier investigations have shown that under stress conditions, the survival of the cells depends strongly on maintaining or adjusting the activity of VHA^33^. In the light of these observations, it is not surprising that our mutant plants had higher membrane damage as also indicated by electrolyte leakage and MDA levels and a declined activity of VHA.

Our study further revealed a strong positive relationship among *Thielavia, Zopfiella, Taeniolella, Phoma* and *Edenia*, and root to shoot ratio in the wild type variety. Interestingly, the results for *Fusarium* (a notorious root rot causing genus) were not significant for root to shoot ratio but were substantially correlated to yield. This observation is in contradictory with those reported from previous studies where the abundance of *Fusarium* sp. was positively correlated with yield decline^16,19^ or in other cases with disease incidence^20^. From these observations we therefore infer that wild type samples attracts non-pathogenic group of *Fusarium* species.

In the current study the most prevalent species of *Cladosporium* was *Cladosporium cladosporioides*, which inhabits senescent plant parts^53^. Moreover, a recent study has indicated the ability of *C. cladosporioides* as a new biological control agent of chrysathemum white rust^54^. Interestingly, quantification analysis by RT-PCR further confirmed a substantial increase in C. *cladosporioides* in the rhizosphere soil of *esl* mutant than the wild type variety (Supplementary Fig. 3).

## Conclusion

Taken together, our results demonstrated that the *esl* genotype exhibited reduced biomass and overall yield as compared to the wild type variety probably because of reduced cell membrane integrity, increased chlorophyll degradation, and greater membrane damage. We propose that plant survival during senescence like any other stresses relies on the activity of VHA and maintaining a lower K^+^ efflux, H^+^ and increasing Ca^2+^ influx. From our study it is evident that VHA-A1 gene mutation indeed influenced microbial composition and diversity in rhizosphere soil favoring an abundance of certain group of microbes and suppressing others. Based on these outcomes, additional work is recommended to characterize the roles of *esl* mutant rice root exudates in shaping rhizospheric soil microbial community and structure. Moreover, the isolation of specific fungi (e.g. *Penicillium* spp. and *Cladosporium* spp.) and their relationships with early senescence in rice would also help to understand this global ecological and agricultural challenge for a sustainable agriculture.

## Materials and Methods

### Plant materials and treatments

The experiments were carried out at the Experimentation Station of Fujian Agriculture and Forestry University, Fuzhou, Fujian, China (119.280 E, 26.080N) during the rice growing season (May–September). Two rice genotypes, an *indica* rice cultivar (Fu142) and its corresponding mutant with the *esl* phenotype were used in this study. The *esl* mutant was a resultant from Fu 142 cultivar (*Oryza sativa* L. ssp. *indica*) mutated by gamma-irradiation of Fu142 mature seeds. A steadily inherited mutant was attained by successive self-pollination and the identification of the plant phenotype in the M2–M7 generations^5^. The seeds were sown in the nursery field and then seedlings were transplanted at 5-leaf stage with a spacing of 0.18 × 0.18 m and one seedling per hill. Each plot was 4 m × 4 m that received 225 kg N·ha^−1^ (60% as basal-dressing and 40% applied during panicle initiation stage), 67.5 kg P_2_O_5_·ha^−1^ (as the basal dressing) and 180 kg K_2_O·ha^−1^ (50% as the basal dressing and 50% as the top dressing). The field plots were arranged in a randomized complete block design with each genotype replicated thrice overtime.

### Soil sampling and determination of chemical properties

Before grain-filling, ten plants of each genotype were randomly tagged, and data were collected from these plants. Six plants from plots of *esl* mutant rice and its isogenic wild type variety were carefully removed, and the rhizosphere soils (about 20 cm rooting depth) were collected by shaking the roots to eliminate small clumps of soil clinging to the roots. Soil adhering to the root system was carefully removed using forceps and mixed thoroughly by passing through a 2-mm sieve to form a single aggregate sample per treatment.

The soil samples were used as soon as possible or stored in a refrigerator at −80 °C until further processing. Selected soil variables measured comprised of total phosphorus (TP by sodium carbonate fusion); total nitrogen (TN by Kjeldahl digestion); and total potassium (TK by NaOH melt flamer)^55^. Available phosphorus (AP) was extracted with hydrochloric acid and ammonium fluoride and determined using molybdenum blue method, and available nitrogen (AN) was measured by alkaline hydrolysable technique, while available potassium (AK) was extracted by ammonium acetate and determined by flame photometry^56^. Soil pH analysis was done at 2.5:1, water: soil ratio and examined with a glass electrode pH meter. All treatments were subjected to the same fertilization and field management throughout the study period.

### Measurements of net Ca^2+^, K^+^, and H^+^ flux with NMT

Net fluxes of Ca^2+^, K^+^, and H^+^ were determined using Non-invasive Micro-Test Technology (NMT) and influxes V2.0 (Younger USA LLC, Amherst, MA 01002, USA) software as described earlier^57^. The principle of this method and instrument are detailed in Li *et al*.^58^. Rice plants were equilibrated in measuring solution for 10 min, the equilibrated rice plants were transferred to the measuring chamber with a small petri dish (6 cm diameter) containing 10 ml of fresh measuring solution. This was connected to the microelectrode vibrating in the solution between two positions (5 and 35 µm, respectively) from the root surface, along an axis vertical to the root apex. Then the background was recorded by vibrating the electrode in measuring solution without roots. After backfilling, electrode tip was filled with a commercially available ion-selective mixture (Ca^2+^, K^+^, and H^+^). Flux records were constantly made every 6 seconds in the apical regions. All measurements of fluxes were carried out at Fujian Provincial Key Laboratory of Agroecological Processing and Safety Monitoring, Fujian, China.

### Determination of physiological and biochemical parameters

Chlorophyll content was determined from fresh intact leaves in terms of SPAD values using a portable optical meter (*SPAD* 502, *Konica-Minolta*, Osaka, Japan). The level of lipid peroxides was measured in terms of malondialdehyde (MDA) accumulation and expressed as 2-thiobarbituric acid (TBA) reactive metabolites, as formerly described by Nahar *et al*.^39^. In addition, root oxidizability was determined by 2,3,4-Triphynal terazolium chloride [TTC] reduced method by Zhou *et al*.^42^. Moreover, the (membrane damage) electrolyte leakage in the roots and leaves was determined according to Ning *et al*.^59^ and expressed as the percentage of the total conductivity. The activity of VHA was calorimetrically evaluated by measuring the release of phosphate (Pi) and expressed in µmol Pi mg^−1^ protein h^−1^. In brief, root and leaf membrane proteins were extracted and 10 µg microsomal membranes were incubated for 40 min at 28 °C. Finally, 40 mM citric acid stop solution was added to the reactions, while 10 µg bovine serum albumin was used as blank^30^. The activity of VHA was assayed as described earlier by Zhang *et al*.^60^.

### DNA extraction and purification

Genomic DNA was extracted from 0.5 g soil subsamples using Biofast Soil Genomic DNA Extraction Kit (Bioer Technology Co., Ltd., Hangzhou, China) in accordance with the manufacturer’s guidelines. Three sequential DNA extractions of each sample were combined before PCR (to minimize DNA extraction bias). We then used Nanodrop spectrophotometer (Thermo Scientific, Wilmington, DE) to quantify the extracted DNA and stored at −20 °C for further molecular investigation. The fungal hypervariable regions V4-V9 of the 18S rRNA gene were PCR-amplified using independently bar-coded forward primers 528F, 5′-GCATCGATGAAGAACGCAGC-3′ and reverse primer 706R, 5′-TCCTCCGCTTATTGATATGC-3′.

### PCR purification and pyrosequencing

Amplicon products were examined on 2% agarose gel and samples with bright main strip between 400–450 bp were chosen for additional experiments. PCR products were mixed in equimolar ratios and purified with Qiagen Gel Extraction Kit (Qiagen, Hilden, Germany). Sequencing libraries were then generated using TruSeq DNA PCR-Free Sample Preparation Kit (Illumina, San Diego, USA) following the manufacturer’s protocol. Pyrosequencing was finally performed on an Illumina HiSeq. 2500 platform, which generated 250 bp paired-end reads.

### OTU (Operational Taxonomic Unit)-based sequence analysis

Sequences were trimmed and allotted to different samples based on their exclusive barcodes. Paired-end reads were united using FLASH (Baltimore, MD, USA)^61^. After quality cleaning and chimera elimination, the remaining sequences were denoted as effective tags and used to perform OTU cluster and species annotation. Sequences with less than 3% variation were assigned to the same OTU through UPARSE software (Uparse v7.0.1001; CA, USA). Species annotation was carried out by the Unite Database (https://unite.ut.ee/) based on Blast algorithm which was calculated according to Quantitative Insights Into Microbial Ecology (QIIME V 1.7.0; CO, USA). Representative sequence for each OTU was screened for further annotation.

The taxonomic identity of the fungi was determined using the RDP software^62^ and Silva database. Sequences were also assigned to phylotypes using MUSCLE software^63^, followed by normalization using a standard of sequence number corresponding to the sample with the least sequences. Subsequently, analysis of alpha and beta diversity was performed based on the output normalized data.

### Real time PCR for *Cladosporium cladosporioides*

The fragments of *Cladosporium cladosporioides* were cloned into pEASY-T1 simple vector (TransGen Biotech Co., Beijing, China) and two plasmids were purified. After determining DNA concentration, ten-fold serial dilutions (10^−1^ to 10^−9^) was followed to construct standard curve using log10 value against the threshold cycle (Ct) value. The reaction of standard curve was performed following the RT-PCR amplification procedure as described in Supplementary Table 8. We then performed Real-time PCR quantifications of *C. cladosporioides* (primer sets (18S-F: TTGTCCGACTCTGTTGCCTC and 18S-R: CGCTTAGGGGACAGAAGACC) in six soil samples. Reaction was performed in 15 μl mixture, containing 7.5 μl TransStart Green PCR SuperMix (Transgen Biotech Co., Beijing, China), 0.6 μl of each primer (10 μM) and 20 ng DNA.

### Bioinformatics and statistical analysis

To compute Alpha Diversity, we rarified the OTU table and calculated six metrics: Observed-species, Chao1, Shannon, Simpson, abundance based coverage (ACE) and Good-coverage, which were calculated with QIIME (ver. 1.7.0) and illustrated with R software (ver. 2.15.3). Simpson and Shannon indices were used to examine the community diversity, while the abundance of each fungal taxa, from phylum to species level, and was depicted graphically using Krona Chart. To compare the fungal community structures across all samples based on the OTU composition β-diversity analysis was calculated using both unweighted and weighted Unifrac distance and Principal Coordinate Analysis (PCoA). Moreover, PCoA based on pairwise Bray-Curtis dissimilarity was performed using R (version 2.15.3) with WGCNA package and *ggplot2* (Elegant graphics for data analysis, New York, NY, USA). In addition, Heat map figures were generated using custom R scripts. Venn diagrams were constructed to illustrate the shared fungal communities among sample groups and to create essential microbiome in the rhizosphere, while NMT figures were constructed with Graphpad prism 5 and Origin 8.0 software. For all parameters, multiple comparison was performed by one-way analysis of variance (ANOVA) followed by Tukey’s test (P ≤ 0.05) by Statistix version 8.1 software. Pearson’s correlation analysis was performed for abundant fungi, yield, yield related traits and physiological traits by SPSS 22.0 software, while the histograms were created using Microsoft Excel 2013.

## Electronic supplementary material

supplementary information
