# Supplementary material for: Gene mutation associated with esl mediates shifts on fungal community composition in rhizosphere soil of rice at grain-filling stage
Source: Sci Rep. 2018 Nov 30;8:17521. doi: 10.1038/s41598-018-35578-y (PMC6269515; doi:10.1038/s41598-018-35578-y)
Supplement: Supplementary file 1 — supplementary information [file 41598_2018_35578_MOESM1_ESM.docx]

**Gene mutation associated with *esl* mediates shifts on fungal community composition in rhizosphere soil of rice at grain-filling stage**

Puleng Letuma ^1,2,4^, Yasir Arafat^2,3^, Muhammad Waqas^1,2^, Feifan Lin^2,3^, Weiwei Lin^2,3^, Yiyang Zhang^1,2^, Mamello Masita^3,4^, Kai Fan^2,3^ , Zhaowei Li^2,3**^ & Wenxiong Lin^1,2,3**^

1 College of Crop Science, Fujian Agriculture and Forestry University, Fuzhou 350002, Fujian, China, Key Laboratory for Genetics, Breeding and Multiple Utilization of Crops, Ministry of Education

2 Fujian Provincial Key Laboratory of Agroecological Processing and Safety Monitoring, Fujian Agriculture and Forestry University, Fuzhou, China

3 College of Life Sciences, Fujian Agriculture and Forestry University, Fuzhou, China

4 Crop Science Department, Faculty of Agriculture, National University of Lesotho, Roma 180, Lesotho

***** Correspondence: lizw197@163.com (Z.L); lxw@fafu.edu.cn (W.L.); Tel.: +86-0591-837-789301 (S.L. & W.L.)


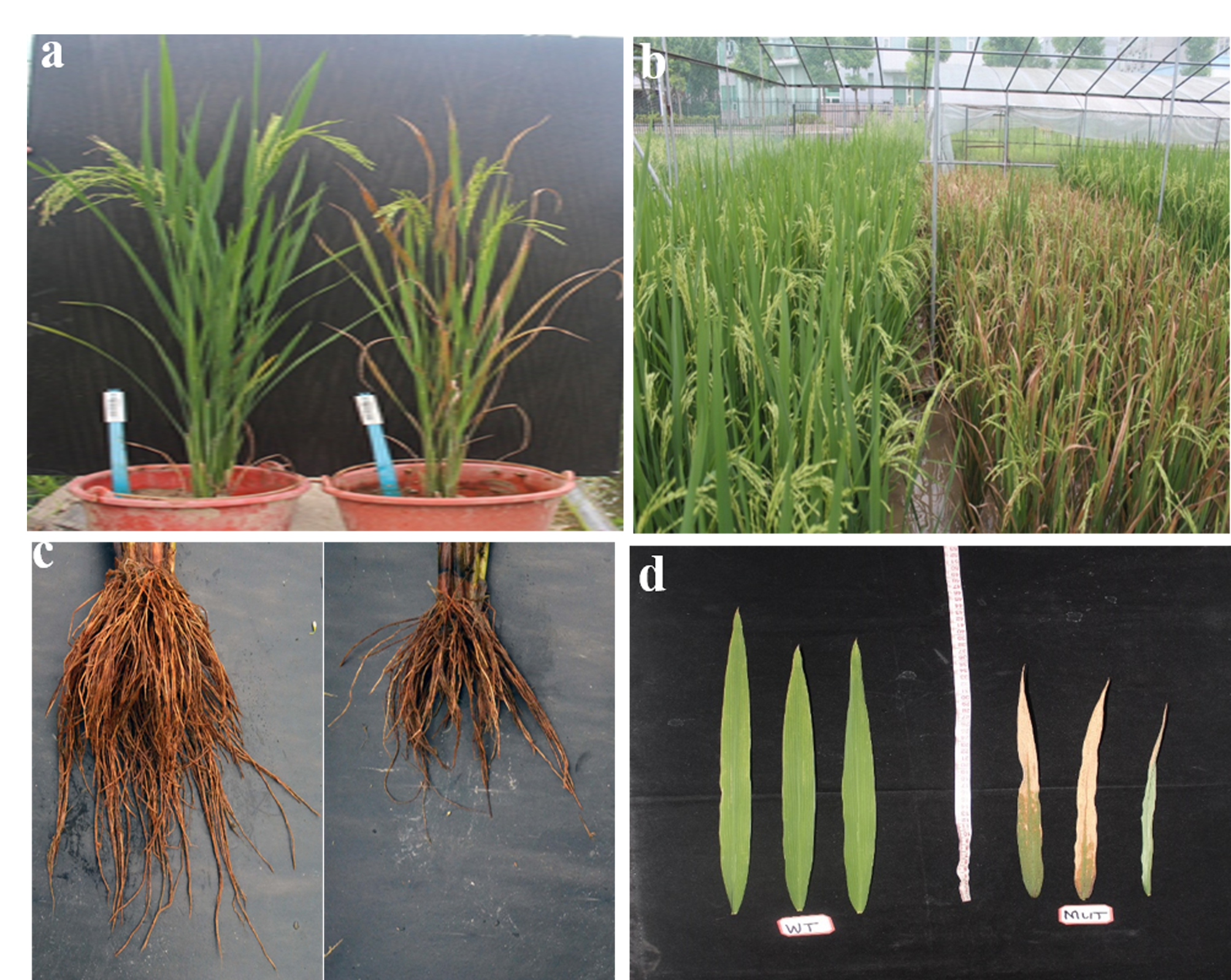


**Supplementary Fig 1**. Above ground and below ground parts for an *esl* mutant rice (MUT) and its wild type variety (WT) (a) above ground picture of the two genotypes *(*WT *on left*, MUT on the *right*); (b) wild type variety (*on the left*) and *esl* mutant rice (*on the right*) growing in the field; (c) root of wild type variety (*on the left)* and root of *esl* mutant rice (*on the right*); d) leaves of the wildtype (on the *left*) and esl mutant on the (*right).*


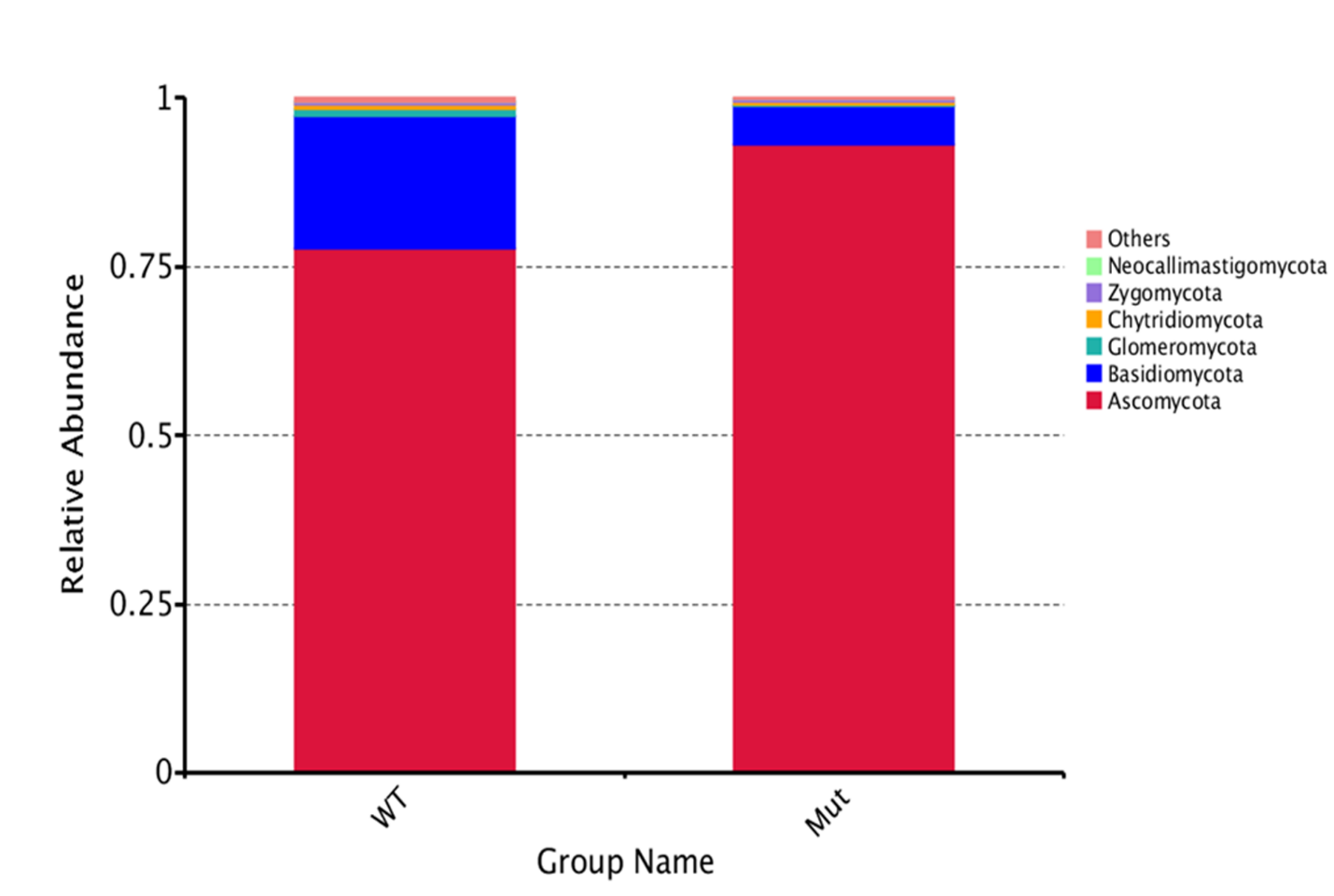


**Supplementary Fig 2.** Relative abundance of the fungal phyla in the rhizosphere soils of *esl* mutant rice (Mut) and it’s wild type (WT) variety


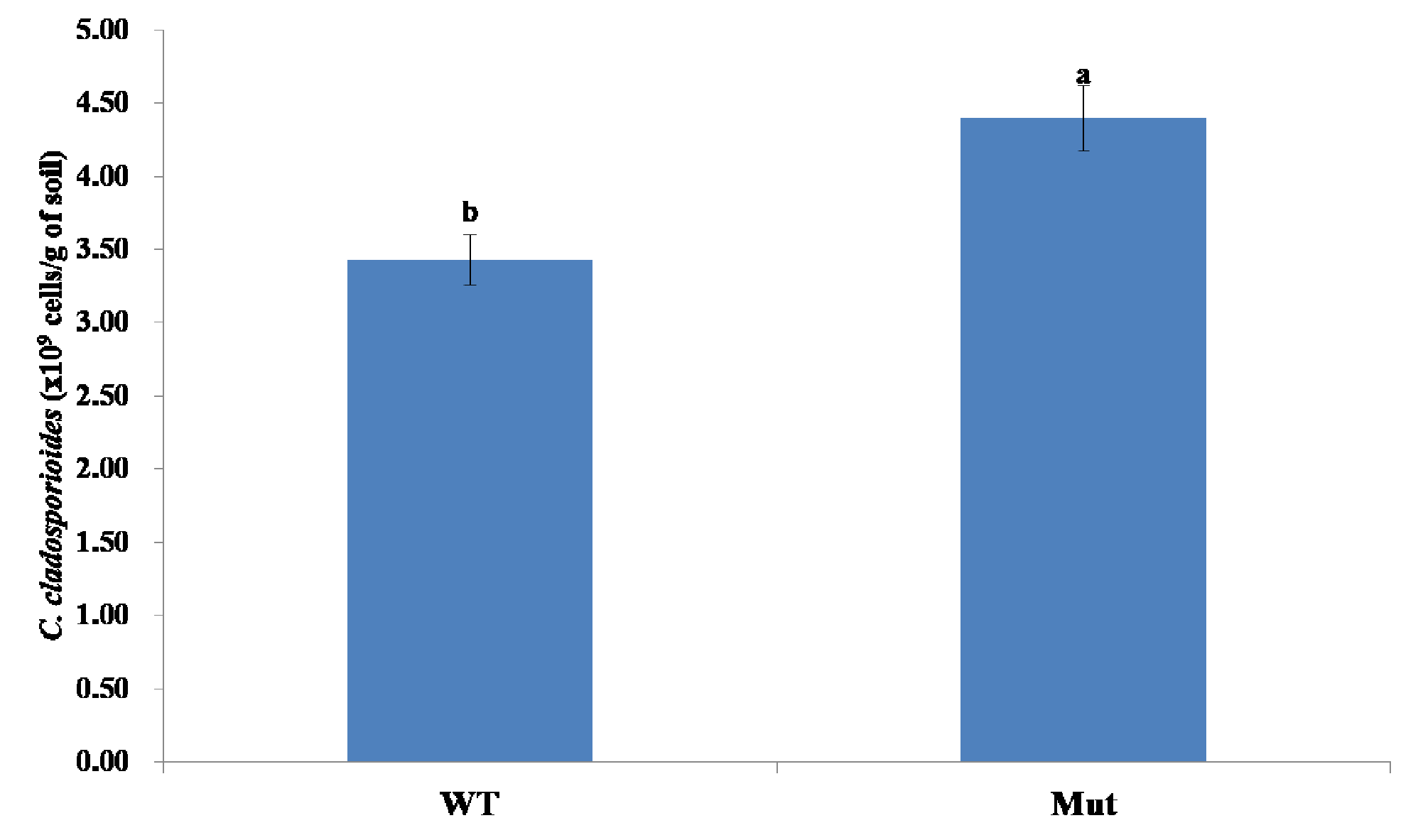


**Supplementary Fig 3.** Quantification through RT-PCR for *Cladosporium cladosporioides* in soil samples of *esl* mutant rice and its wild type variety at grain-filling stage

| **Supplementary Table 1.** Soil chemical properties from an *esl* mutant rice and it’s wild type variety at grain-filling stage | | |
| --- | --- | --- |
| **Soil chemical properties** | **Wild type** | **Mutant** |
| Total nitrogen (g/kg) | 1.0563a | 1.0528a |
| Available Nitrogen (mg/kg) | 14.683a | 14.650a |
| Total Phosphorus [TP] (g/kg) | 26.718a | 26.652a |
| Available phosphorus [AP] (mg/kg) | 101.67a | 95.00a |
| Total Potassium [TK] (g/kg) | 11.327a | 11.312a |
| Available Potassium [AK] (mg/kg) | 134.34a | 131.65a |
| pH | 5.4467a | 4.89a |

| **Supplementary Table 2**. Summary of sequencing data for an early senescence rice mutant and its wild type variety at grain-filling stage | | | | | | | | | |
| --- | --- | --- | --- | --- | --- | --- | --- | --- | --- |
| Sample Name | Raw PE(#) | Combined(#) | Qualified(#) | Nochime(#) | AvgLen(nt) | Q20 | Q30 | GC% | Effective% |
| Mut1 | 37,798 | 37,290 | 36,275 | 35,682 | 324 | 99.07 | 98.03 | 55.15 | 94.04 |
| Mut2 | 50,340 | 49,586 | 48,231 | 45,707 | 319 | 99.13 | 98.15 | 54.53 | 90.8 |
| Mut3 | 55,457 | 54,639 | 53,179 | 52,413 | 324 | 99.09 | 98.04 | 54.91 | 94.51 |
| WT1 | 61,195 | 60,140 | 58,577 | 57,514 | 325 | 99.02 | 98.08 | 52.19 | 93.98 |
| WT2 | 57,723 | 56,697 | 54,973 | 54,032 | 326 | 99.05 | 98.13 | 52.27 | 93.61 |
| WT3 | 63,548 | 62,408 | 60,529 | 59,541 | 325 | 99.06 | 98.13 | 52.3 | 93.69 |
| TOTAL | **326,061** | **320,760** | **311,764** | **304,889** |  |  |  |  |  |

| **Supplementary Table 3**. Summary of total tag, taxon tag, unique tags and OTU numbers for different soil samples | | | | |
| --- | --- | --- | --- | --- |
| **Sample Name** | **Total tag** | **Taxon Tag** | **Unique Tag** | **OUT num** |
| WT1 | 57514 | 57244 | 270 | 861 |
| WT2 | 54032 | 53777 | 255 | 884 |
| WT3 | 59541 | 59258 | 283 | 868 |
| Mut1 | 35682 | 35520 | 162 | 556 |
| Mut2 | 45707 | 45297 | 410 | 674 |
| Mut3 | 52413 | 52156 | 257 | 720 |
|  | **304889** | **303252** | **1637** | **4563** |

| **Supplementary Table 4.** Relative abundance of fungal phylum identified in soil samples | | | | |
| --- | --- | --- | --- | --- |
| **Taxonomy** | **Total Count** | **Total %** | **WT %** | **MUT %** |
| *Ascomycota* | 5 | 85.50% | 77.77 | 93.17 |
| *Basidiomycota* | 1 | 12.60% | 19.63 | 5.67 |
| *Chytridiomycota* | 0 | 0.50% | 0.57 | 2.3 |
| *Glomeromycota* | 0 | 0.60% | 1.07 | 0.17 |
| *Neocallimastigomycota* | 0 | 0.00% | 0.00 | 0.00 |
| *Zygomycota* | 0 | 0.40% | 0.47 | 0.32 |
| No blast hit;Other | 0 | 0.30% | 0.5 | 0.23 |

**Supplementary Table 5**. Pearson’s correlation analysis for abundant genera, biomass and yield related traits for wild type variety and *esl* mutant

| **Parameter/genera** | **R/S** | **Panicle Numbers** | **Total grains** | **1,000 seed weight** | **% filled grain** | **filled grain numbers** |
| --- | --- | --- | --- | --- | --- | --- |
| *Cladosporium* | -.872* | -.901* | -.842* | -.926** | -.874* | -.859* |
| *Thielavia* | .907* | .976** | .944** | .979** | .990** | .958** |
| *Zopfiella* | .899* | .978** | .948** | .979** | .988** | .962** |
| *Gibberella* | -.904* | -.951** | -.895* | -.933* | -.923** | -.912* |
| *Taeniolella* | .912* | .978** | .943** | .982** | .990** | .958** |
| *Fusarium* | .765 ^ns^ | .932** | .942** | .915* | .898* | .942** |
| *Westerdykella* | .702^ns^ | .779 ^ns^ | .786 ^ns^ | .755 ^ns^ | .859* | .794 ^ns^ |
| *Hongkongmyces* | .688^ns^ | .733 ^ns^ | .727 ^ns^ | .714 ^ns^ | .829* | .740 ^ns^ |
| *Talaromyces* | -.269 ^ns^ | -.225 ^ns^ | -.300 ^ns^ | -.333^ns^ | -.296 ^ns^ | -.122 ^ns^ |
| *Phoma* | .897* | .968** | .925** | .965** | .983** | .946** |
| *Edenia* | .941** | .954** | .907* | .971** | .995** | .925** |
| *Humicola* | -.961** | -.871* | -.782 ^ns^ | -.902** | -.968** | -.817* |
| *Penicillium* | -.906* | -.881* | -.825* | -.971** | -.881* | -.836* |

** Significant at 1 % level of probability, * Significant at 5 % level of probability, ns non-significant R/S= root to shoot ratio

| **Supplementary Table 6**. Correlation analysis of abundant genera with physiological parameters for wild type variety and *esl* mutant | | | | | | | | |
| --- | --- | --- | --- | --- | --- | --- | --- | --- |
| **Trait/Genera** | **VHA activity (r) (µmol pi mg^-1^ protein h^-1^)** | **VHA activity (l) (µmol pi mg^-1^ protein h^-1^** | **Tchl** | **MDA (r) (nmol g^-1^ FW)** | **MDA(l) (nmol g^-1^ FW)** | **E.L (%) r** | **E.L (%) l** | **% R.O.** |
| *Cladosporium* | -.833^*^ | -.947^**^ | -.961** | .964** | .824* | .895* | .849* | -.757 |
| *Thielavia* | .979^**^ | .955^**^ | .956** | -.970** | -.983** | -.999** | -.983** | .900* |
| *Zopfiella* | .983^**^ | .942^**^ | .936** | -.960** | -.991** | -.996** | -.988** | .907* |
| *Gibberella* | -.893^*^ | -.973^**^ | -.970** | .989** | .896* | .943** | .914* | -.823* |
| *Taeniolella* | .976^**^ | .959^**^ | .967** | -.979** | -.975** | -.999** | -.977** | .890* |
| *Fusarium* | .940^**^ | .884^*^ | .799 | -.849* | -.981** | -.924** | -.978** | .964** |
| *Westerdykella* | .877^*^ | .684 | .699 | -.707 | -.878* | -.845* | -.847* | .785 |
| *Hongkongmyces* | .838^*^ | .622 | .647 | -.663 | -.842* | -.804 | -.802 | .722 |
| *Talaromyces* | -.111 | -.442 | -.346 | .374 | .138 | .189 | .204 | -.236 |
| *Phoma* | .983^**^ | .894^*^ | .923** | -.954** | -.969** | -.983** | -.961** | .853* |
| *Edenia* | .955^**^ | .952^**^ | .958** | -.968** | -.969** | -.994** | -.965** | .862* |
| *Humicola* | -.888^*^ | -.847^*^ | -.941** | .936** | .868* | .937** | .852* | -.678 |
| *Penicillium* | -.811 | -.971^**^ | -.877* | .912* | .895* | .894* | .903* | -.835* |

* *Significant at 1 % level of probability, * Significant at 5 % level of probability: Tchl, Total chlorophyll; MDA (r), (l), Malondialdehyde in roots and leaves, respectively; E.L. % (r), (l), electrolyte leakage percentage in roots and leaves respectively; % R.O., percentage root oxidizability; VHA activity (r), (l), vacuolar H^+^-ATPase activity in roots and leaves, respectively.

| **Supplementary Table 7.** Correlation analysis of biomass, yield and physiological traits determined for wild type variety and *esl* mutant | | | | | | |
| --- | --- | --- | --- | --- | --- | --- |
| **Parameter** | **R/S** | **panicle Numbers** | **Total grains** | **1,000-seed weight** | **% filled grain** | **filled grain numbers** |
| Tchl | .890* | .936** | .894* | .915* | .952** | .912* |
| % R.O. | .674^ns^ | .922** | .966** | .781^ns^ | .836* | .952** |
| MDA (r) | .919** | .973** | .917* | .972** | .961** | .937** |
| MDA (l) | -.869* | -.968** | -.948** | -.916* | -.962** | -.957** |
| E.L. % (r) | -.905* | -.975** | -.944** | -.939** | - .992** | -.959** |
| E.L. % (l) | -.848* | -.981** | -.970** | -.914* | -.953** | - .976** |
| VHA activity (r) | .871* | .952* | .927* | .892* | .920** | .932** |
| VHA activity (l) | .823* | .977* | .969* | .920* | .963** | .979** |

| **Supplementary Table 8.** Taxon-specific primer sets and their thermal cycling conditions for RT-PCR. | | | |
| --- | --- | --- | --- |
| **Primer** | **Sequence (5´- 3´)** | **Thermal conditions** | **Reference** |
| 18S-F  18s-R | TTGTCCGACTCTGTTGCCTC, CGCTTAGGGGACAGAAGACC | 95 for 3min,95 for 30s, 55^0^C for 30s, 72 for 5min, 35 cycles | Lievans et al., 2005 |

Lievens, B., Brouwer, M., Vanachter, A.C., Lévesque, C.A., Cammue, B.P., and Thomma, B.P. (2005). Quantitative assessment of phytopathogenic fungi in various substrates using a DNA macroarray. Environ. Microbiol. 7, 1698-1710. doi: 10.1111/j.1462-2920.2005.00816.x
